# Pre-clinical studies of bone regeneration with human bone marrow stromal cells and biphasic calcium phosphate

**DOI:** 10.1186/scrt504

**Published:** 2014-10-13

**Authors:** Meadhbh Á Brennan, Audrey Renaud, Jérôme Amiaud, Markus T Rojewski, Hubert Schrezenmeier, Dominique Heymann, Valerie Trichet, Pierre Layrolle

**Affiliations:** INSERM, UMR957, Laboratory of the Pathophysiology of Bone Resorption, Faculty of Medicine, University of Nantes, 1 Rue Gaston Veil, 44035 Nantes, France; Institute for Clinical Tranfusion Medicine and Immunogenetics, German Red Cross Blood Donor Service, Ulm, Germany

## Abstract

**Introduction:**

Repair of large bone defects remains a significant clinical challenge. Bone marrow stromal cells (BMSCs), a subset of which is known as bone marrow-derived mesenchymal stem cells, show therapeutic potential for bone regeneration. However, their isolation, expansion and implantation will need to be conducted under good manufacturing practices (GMP) at separate locations. An investigation which mimics this clinical scenario where large bone defects shall be regenerated is required before clinical trials can be initiated.

**Methods:**

Seven batches of 100 million human *ex*-*vivo* expanded BMSCs from five donors were transported fresh in syringes from a GMP facility in Germany to France. BMSCs were mixed with biphasic calcium phosphate (BCP) biomaterial prior to subcutaneous implantation in nude mice. The capacity of BMSCs in unison with BCP to regenerate critical sized cranial bone defects was also evaluated. BMSCs expressing luciferase were used to assess the viability and bio-distribution of implanted cells. *In situ* hybridization, using the human-specific repetitive *Alu* sequence, was performed for the identification of human cells in explants.

**Results:**

Eight weeks after implantation of BMSCs, mineralized bone containing mature bone marrow territories was formed in ectopic sites and in calvaria defects. Significant loss of cell viability was observed by bioluminescence imaging and only 1.5 percent of the initial number of transplanted cells remained after 37 days. After eight weeks, while explants were comprised primarily of host cells, there were also human cells attached along the periphery of BCP and embedded in osteocyte lacunae dispersed throughout the newly formed bone matrix.

**Conclusions:**

This study demonstrates the safety and efficacy of BMSC/BCP combinations and provides crucial information for the implementation of BMSC therapy for bone regeneration.

## Introduction

Successful repair of bone defects caused by trauma, cancer or metabolic diseases remains a significant clinical challenge for reconstructive surgeons. Bone is the most frequently transplanted tissue, with 2.2 million bone replacement procedures conducted globally each year [1]. Autologous bone transplantation is limited by the quantity and quality of grafted bone and can lead to complications at the second surgical site, while allogenic bone grafts pose the risk of disease transfer and immunologic rejection. Consequently, there are considerable incentives for developing alternative solutions for bone regeneration.

Significant opportunities exist for tissue engineering strategies in orthopedic and maxillofacial surgery. Synthetic biomaterial scaffolds in association with bone marrow stromal cells (BMSCs), a subset of which is known as bone marrow-derived mesenchymal stem cells, could overcome the limitations of biological bone grafts. BMSCs are multipotent progenitor cells, capable of differentiating into osteoblasts, chondrocytes and adipocytes [2], and are therefore considered promising for tissue engineering applications. Human BMSCs can be isolated from a small volume of bone marrow aspiration under local anesthesia. However, due to the diminutive number of BMSCs in bone marrow (0.001 to 0.01% of bone marrow mononuclear cells (BM-MNCs)) [3], expansion *ex vivo* is necessary to obtain clinically transplantable doses. Since BMSCs are deemed an advanced therapy medicinal product by the European Commission [4], they must be produced in accordance with good manufacturing practice (GMP). Safe, robust and GMP-compliant protocols for large-scale isolation and expansion of BMSCs, which avoid animal products such as fetal calf serum by using human platelet lysate (PL), have been developed [5–8]. Published data identified transforming growth factor beta-1, vascular endothelial growth factor, platelet-derived growth factor, fibroblast growth factor and epidermal growth factor among effectors of PL activity [5, 9]. Furthermore, it has been demonstrated previously that PL is a safe alternative to fetal calf serum for culturing human BMSCs and that it favors both osteoblastic differentiation and bone tissue formation [6, 10].

The capacity of BMSCs for bone repair has been studied *in vivo* with promising results [11–13]. However, for clinical relevance it is clear that the isolation, expansion and implantation of cells will need to be conducted at separate facilities, often with considerable distances between the cell production site and the surgical room. Cryopreserved BMSCs maintain their bone formation capabilities [14]. However, the transportation of frozen cells directly to the operating theater is not feasible because of the time required for cells to recover function after thawing [15] and the potential adverse effects of the cryoprotectants [16]. Veronesi and colleagues have recently determined that when freshly harvested BMSCs are suspended in a saline/human serum albumin (HSA) solution, cell viability is maintained and bone formation in small-scale implants can be achieved [17]. Nevertheless, there is a need to evaluate the bone regeneration of BMSCs that have undergone large-scale GMP expansion and transportation to a separate facility in clinically relevant numbers and time frames.

Determining the cell dose of BMSCs required for adequate bone and hematopoiesis formation is of immense interest for bone tissue engineering. While it might be expected that higher numbers of cells would lead to increased bone formation, Mankani and colleagues have demonstrated a threshold beyond which more transplanted cells do not lead to more bone formation [12].

Adequate biomaterial scaffolds are required for the transplantation of BMSCs targeted at repairing osseous defects. BMSCs combined with porous calcium phosphate ceramics, namely hydroxyapatite/beta-tricalcium phosphate, have been shown to induce bone formation in the subcutis of nude mice [12, 18, 19] and in femoral defects in rats [20]. Biphasic calcium phosphate (BCP) biomaterials are widely used for bone augmentation, for filling bone defects in combination with autologous bone marrow, or for supplementing autologous bone grafts.

A further requirement for an advanced therapy medicinal product prior to entering clinical trials is the demonstration of the bio-distribution of the transplanted BMSCs. There are conflicting observations as to the fate of BMSCs transplanted within biomaterial scaffolds and whether the newly formed bone is of host or donor cell origin. Evidence for donor BMSCs being involved in bone regeneration comes from studies that demonstrate osteoblasts and osteocytes of donor origin within newly formed bone [13, 21, 22]. Less donor cells were present in later harvest transplants than at earlier time points, however [22]. Conversely, Tasso and colleagues demonstrated that donor BMSCs disappeared from the scaffold after the second week of implantation and the newly formed bone was entirely of host origin [23]. Studies assessing implanted cell survival have shown substantial death of implanted donor cells [24, 25]. One study revealed that 50% of initial donor BMSCs remained 48 hours after implantation, while 5% remained after 8 weeks [24]. Another study showed that less than 1% was detectable after 30 days [25]. An investigation of the real-time metabolic activity of transplanted cells and the cell origin of bone formation would therefore shed light on whether bone is formed directly by donor cells, or whether a paracrine effect induces a host stromal cell response.

The first objective of this study was to determine the optimal cell dosage required for bone formation before scaling up to relevant clinical numbers of BMSCs. The study sought to transport freshly produced BMSCs from a GMP facility, where they were isolated and expanded, to another site where they were mixed with biomaterial and implanted, mimicking as much as possible the clinical scenario where large bone defects of 5 to 10 cm^3^ shall be regenerated. The objectives were to quantify the degree of ectopic bone and hematopoietic compartment formation, as well as bone regeneration in critical-sized defects in nude mice. A final premise of this study was to explore the cell fate and biological activity of transplanted human BMSCs and to determine whether newly formed bone was of host or donor origin.

## Methods

### Biomaterial

BCP, a ceramic composed of hydroxyapatite/beta-tricalcium phosphate in a ratio of 20/80 by weight, was used because this composition has previously demonstrated bone induction [19]. BCP granules, ranging in size from 1 to 2 mm, were supplied by Biomatlante (Vigneux de Bretagne, France) under the brand name MBCP+. MBCP + is CE and US Food and Drug Administration 510(k) approved as a synthetic bone substitute. BCP discs, 7 mm in diameter and 2 mm in thickness, were used for calvaria regeneration. The overall porosity (%vol) was 75 ± 5%, with a pore size distribution of 70% (0 to 10 μm), 20% (10 to 100 μm) and 10% (100 to 300 μm). The BCP biomaterials were supplied in double-sealed packaging and gamma-sterilized at 25 kGray.

### Isolation, expansion and characterization of clinical batches of GMP-grade BMSCs

Bone marrow aspirates were obtained from the iliac crest, by standard puncture and aspiration, of healthy human donors (21 to 26 years old) after receiving informed consent according to the Declaration of Helsinki. The project was approved by the Ethical Committee of Ulm University. A maximum of 37 ml bone marrow was aspirated using up to four 20 ml Omnifix syringes (B. Braun, Melsungen, Germany) and transplant aspiration needles (Somatex, Teltow, Germany). Each syringe was prefilled with 1,000 IU heparin in 2 ml NaCl (B. Braun). BMSCs used in this study are of GMP grade and were expanded according to previously published protocols [6]. In brief, BMSCs were isolated from heparinized bone marrow aspirates by seeding 50,000 white blood cells/cm^2^ on two-chamber CellStacks (Corning/VWR, Ulm, Germany) in alpha minimum essential medium (Lonza, Basel, Switzerland) supplemented with 5% GMP-grade human platelet lysate (IKT, Ulm, Germany) in order to avoid animal products [5]. Cells were cultured for 10 or 14 days with medium exchange twice per week. Cells were detached and reseeded at a density of 4,000 BMSCs/cm^2^ on two-chamber CellStacks in alpha minimum essential medium supplemented with 8% PL for a further 5 or 7 days. A production license for this protocol has been granted from the regional government (Regierungspräsidium Tübingen, Germany; production and import license: DE_BW_01_MIA_2013_0040/DE_BW_01_IKT Ulm). To assess the quality of the aspirates, measures such as colony-forming units (CFUs-F), BM-MNC content and doubling times were measured. For phenotypic characterization, flow cytometry was performed as described previously [5, 6]. Fluorescent intensities of 50,000 to 100,000 BMSCs were acquired. Briefly, BMSCs were stained for 15 minutes in 100 μl phosphate-buffered saline using the following combinations of antibodies: IgG-FITC (clone X40), IgG-PE (clone X40), IgG-PerCP (clone X40); CD90-FITC (clone 5E10), CD34-PE (clone 8G12), CD45-PerCP (clone 2D1); CD105-FITC (clone SN6), CD73-PE (clone AD2), CD3-PerCP (clone SK7); and HLA-DR,DQ,DP-FITC (clone Tü39), HLA-A,B,C-PE (clone G46-2.6). All antibodies were sourced from Becton Dickinson (Heidelberg, Germany), except CD105 that was from AbDSerotec (Puchheim, Germany). After washing with phosphate-buffered saline, cells were analyzed using a BD FACScan (BD Biosciences, Heidelberg, Germany).

Differentiation capacity of expanded BMSCs was performed as described previously [5, 6]. Briefly, BMSCs differentiation was induced using adipogenic differentiation medium from Lonza or chondrogenic or osteogenic differentiation media from Miltenyi (Bergisch Gladbach, Germany) according to the manufacturers’ instructions. For detection of adipogenic differentiation, cells were stained with Oil Red O solution in 2-propanol, diluted to 60% using deionized water. Chondrogenic differentiation was detected by Alcian Blue staining, while mineralization was detected by Alizarin red staining.

### Cell numbers required for bone formation

Prior to the transportation of large batches of fresh BMSCs for bone repair, it was necessary to quantify the optimal dose of cells for bone formation in proportionally lower numbers. Different quantities of BMSCs in passage 2 from three different human donors were mixed with 50 mg BCP particles and allowed to attach for 1 hour prior to subcutaneous implantation in nude mice. Number of cells per implant and number of implants per group were as follows: 0 cells, *n* =5; 0.1 × 10^6^ cells, *n* =9; 2 × 10^6^ cells, *n* =9; and 4 × 10^6^ cells, *n* =9.

### Transportation of fresh bone marrow stromal cells

Cells were harvested and washed, and 100 × 10^6^ passage 1 BMSCs from each of the five donors were suspended in 7 ml saline solution supplemented with 4%, 5% or 20% HSA solution (CSL Behring, Hattersheim am Main,Germany) in a sterile luer lock 20 ml syringe (B. Braun). Cells were transported within 24 hours from Ulm (Baden-Wurttemberg, Germany) to Nantes (Pays de la Loire, France) at 20°C via TNT Express overnight courier. Upon arrival, cell viability was confirmed using the trypan blue exclusion method. Each syringe containing BMSCs was connected to a syringe containing 5 cm^3^ BCP particles (2.5 g) using a double luer lock. Cells were allowed to attach to the BCP particles for 1 hour, prior to implantation in nude mice. Cell attachment was confirmed by methylene blue staining.

### Bone marrow stromal cell/biomaterial implantation in the subcutis and calvaria of nude mice

All animal experiments were performed according to Directive 2010/63/UE and after approval of protocols from the local ethical committee (CEEA, Pays-de-la-Loire, France). Immunocompromised female mice (RjOrl: NMRI-*Foxn1*^*nu*^/*Foxn1*^*nu*^) were purchased from a professional breeder (Janvier Labs, Saint-Berthevin, France) at 4 weeks of age. Mice were placed in cages as groups of five in HEPA-filtered closets with water and food *ad libitum,* and were quarantined for a minimum of 10 days prior to surgery. Once under general anesthesia by inhalation of isoflurane, the skin was disinfected with 1% iodine alcoholic solution. For subcutaneous implants, BCP granules alone (BCP) or in association with BMSCs (BCP + BMSCs) were implanted on the dorsal side in two subcutis pockets that were created by skin incisions and tissue dilacerations with sterile instruments. Then 20 × 10^6^ BMSCs (passage 1) with 1 cm^3^ BCP were implanted per subcutaneous site in randomly assigned nude mice. BMSCs from five donors that were transported were implanted subcutaneously: Donor 1, *n* =6; Donor 2, *n* =6; Donor 3, *n* =4; Donor 4, *n* =8; and Donor 5, *n* =7.

For calvaria implants, the mouse was maintained on a stereostatic frame and a skin incision of 1 cm was made to expose the skull. A critical-sized defect 4 mm in diameter was created in the calvaria bone [26] using a trephine and a dental micromotor (Nouvag NM3000; NOUVAG, Goldach, Switzerland). Constant saline irrigation was used during drilling. Then 3 × 10^6^ BMSCs in passage 2 or 3 were seeded onto one side of a BCP disc 1 hour prior to implantation. Cells from three different human donors were used. Discs were overlain cell-side down over the calvaria defect. Calvaria defects overlain with BCP discs alone or defects that were left empty served as controls. Skin incisions were closed with sutures (Filapeau; Peters Surgical, Bobigny, Ile-de-France, France). Analgesic (20 μg/kg; Buprenorphine, Axience, France) was injected intramuscularly before surgery and every 8 hours for 3 days after surgery. Animals were observed daily and body weights were determined weekly. After 4 or 8 weeks, the mice were euthanized by inhalation of an overdose of carbon dioxide gas. Sample sizes for calvaria implantations were as follows: 4 weeks empty, *n* =3; 4 weeks BCP, *n* =3; 4 weeks BCP + BMSCs, *n* =4; 8 weeks empty, *n* =3; 8 weeks BCP, *n* =6; and 8 weeks BCP + BMSCs, *n* =5.

### Decalcified histology preparation, staining and histomorphometry

Explants were observed for signs of tissue necrosis, inflammation or infection, dissected and fixed in 10 volumes of buffered 4% formaldehyde for 72 hours. The skulls were further dissected using a diamond saw. Explants were decalcified in 4.13% ethylenediamine tetraacetic acid/0.2% paraformaldehyde in phosphate-buffered saline, pH 7.4 for 96 hours at 50°C using an automated microwave decalcifying apparatus (KOS Histostation; Milestone Medical, Kalamazoo, Michigan, USA). Samples were then dehydrated in ascending series of ethanol baths (80, 95 and 100%) and finally in butanol for 30 minutes in an automated dehydration station (Microm Microtech, Lyon, France). Samples were then impregnated in liquid paraffin at 56°C (Histowax; Histolab, Gottenburg, Sweden) and embedded at −16°C. Blocks were cut using a standard microtome (Leica RM2255; Leica Biosystems, Nanterre, Ile-de-France, France). Thin histology sections (3 to 5 μm thick) were made perpendicular to the plane of the skin for subcutis implants and in the middle of calvaria defects. Sections were stained by Masson trichrome technique using an automated coloration station (Microm Microtech). This staining combined hematoxylin for cell nuclei (blue/black), fuchsine for cytoplasm, muscle and erythrocytes (red), and light green solution for collagen (green). Stained slices were scanned (NanoZoomer; Hamamatsu, Photonics, Hamamatsu City, Shizuoka Prefecture, Japan) and observed with the virtual microscope (NDP view; Hamamatsu). Histomorphometry of images were processed on the whole implant sections using Image J software (National Institute of Health, Bethesda, Maryland, USA) and the percentage areas of biomaterial, bone tissue, and bone marrow per implant were calculated.

### Nondecalcified histology and backscattered scanning electron imaging

Fixed explants were dehydrated in ascending graded ethanol series followed by one bath of pure acetone and then impregnated with methylmethacrylate for 4 days at 4°C. Each resulting block was cut in half with a circular diamond saw (Leica SP1600) and polished with 4,000-grit silicon carbide sandpaper. Samples were sputtered with a thin layer of gold–palladium (JEOL JFC - 1100E; JEOL, Akishima, Japan) and backscattered electron images were taken using a scanning electron microscope, operating at an accelerating voltage of 15 kV (HITACHI TM-3000; HITACHI, Tokyo, Japan).

### *In situ*hybridization

*In situ* hybridization using the human-specific repetitive *Alu* sequence, which comprises approximately 5% of the total human genome, was performed for identification of human cells as described previously [27] with minor changes. Analysis was performed on ectopic explants of 50 mg BCP particles with or without 2 × 10^6^ of passage 2 BMSCs. Briefly, sections were treated with 3% hydrogen peroxide for 15 minutes at room temperature, then with 10 μg/ml proteinase K (Sigma-Aldrich, Lyon, France) for 10 minutes at 37°C, followed by 0.25% acetic acid in 0.1 M triethanolamine, pH 8.0 for 20 minutes at room temperature. Prehybridization was performed for 3 hours at 56°C in a hybridization buffer containing 4× SSC (Sigma-Aldrich), 50% deionized formamide, 1× Denhardt’s solution, 5% dextran sulfate, 100 μg/ml salmon sperm DNA and molecular-grade water. Hybridization buffer was refreshed with the addition of 70 nM custom DIG-labeled human locked nucleic acid Alu probe 5DigN/5′-TCTCGATCTCCTGACCTCATGA-3′/3DigN (Exiqon, Vedbaek, Denmark) and then target DNA and the probe were denatured for 5 minutes at 95°C. Hybridization was carried out for 19 hours at 56°C. The hybridized probe was detected by immunohistochemistry using biotin-SP-conjugated IgG fraction monoclonal mouse anti-digoxin (Jackson Immunoresearch, West Grove, Pennsylvania, USA) diluted 1/200 in Tris-buffered saline with Tween, 2% bovine serum albumin for 35 minutes at 37°C. Stretoperoxidase was added (1/200 in Tris-buffered saline with Tween) for 45 minutes at 37°C before diaminobenzidine substrate addition (Dako, Les Ulis, Ile-de-France, France). Sections were counterstained with Gill-2 hematoxylin (Thermo Shandon Ltd, Runcorn, UK).

### Bioluminescence imaging of luciferase-expressing BMSCs

To monitor the biological activity of BMSCs implanted subcutaneously, BMSCs were genetically modified using lentiviral units to co-express luciferase and green fluorescent protein (eGFP) reporter genes as described previously [28]. Briefly, cells were amplified in alpha minimum essential medium supplemented with 8% human PL, 100 U/ml penicillin and 100 μg/ml streptomycin, 1 U/ ml heparin and 1 ng/ml basic fibroblast growth factor. The percentage of luciferase and eGFP expressing cells (Luc/eGFP MSCs) was quantified by measuring the eGFP expression by flow cytometry (FC-500 Flow cytometer; Beckman Coulter, Nyon, Switzerland). Luciferase activity was measured for 1 × 10^5^ cells in a 96-well plate with 100 μl lysis substrate buffer (Steady-Glo Luciferase Assay; Promega, Charbonnieres-les Bains, France) and 100 μl culture medium using a VICTOR plate reader (Perkin Elmer, Waltham, Massachusetts, USA). Cells in passage 8 were used for bioluminescence imaging (BLI) experiments. The experimental group consisted of 1 × 10^6^ Luc/eGFP MSCs in unison with 25 mg BCP implanted subcutaneously in nude mice (*n* =6) as described above. A control group consisted of a subcutaneous injection of 1 × 10^6^ Luc/eGFP MSCs on the right dorsal side of mice and 25 mg BCP implanted alone on the left dorsal side (*n* =6). Luciferase activity was monitored at days 0, 2, 4, 8, 11, 14, 18, 21, 28 and 37 using a photon imager (Biospace, Paris, France). Before each acquisition, 3 mg luciferin-D in 250 μl distilled, sterile water was injected intraperitoneally. The BLI results are expressed as counts per minute.

### Statistical analysis

All experiments were blinded and data are expressed as mean ± standard error of the mean. Statistical comparison between groups was performed using a one-way analysis of variance. Minitab statistical software was used (Minitab 16; Minitab Ltd, Coventry, UK). Statistical significance was set as *P* <0.05.

## Results

### Bone marrow stromal cell production and characterization

**Figure 1:**
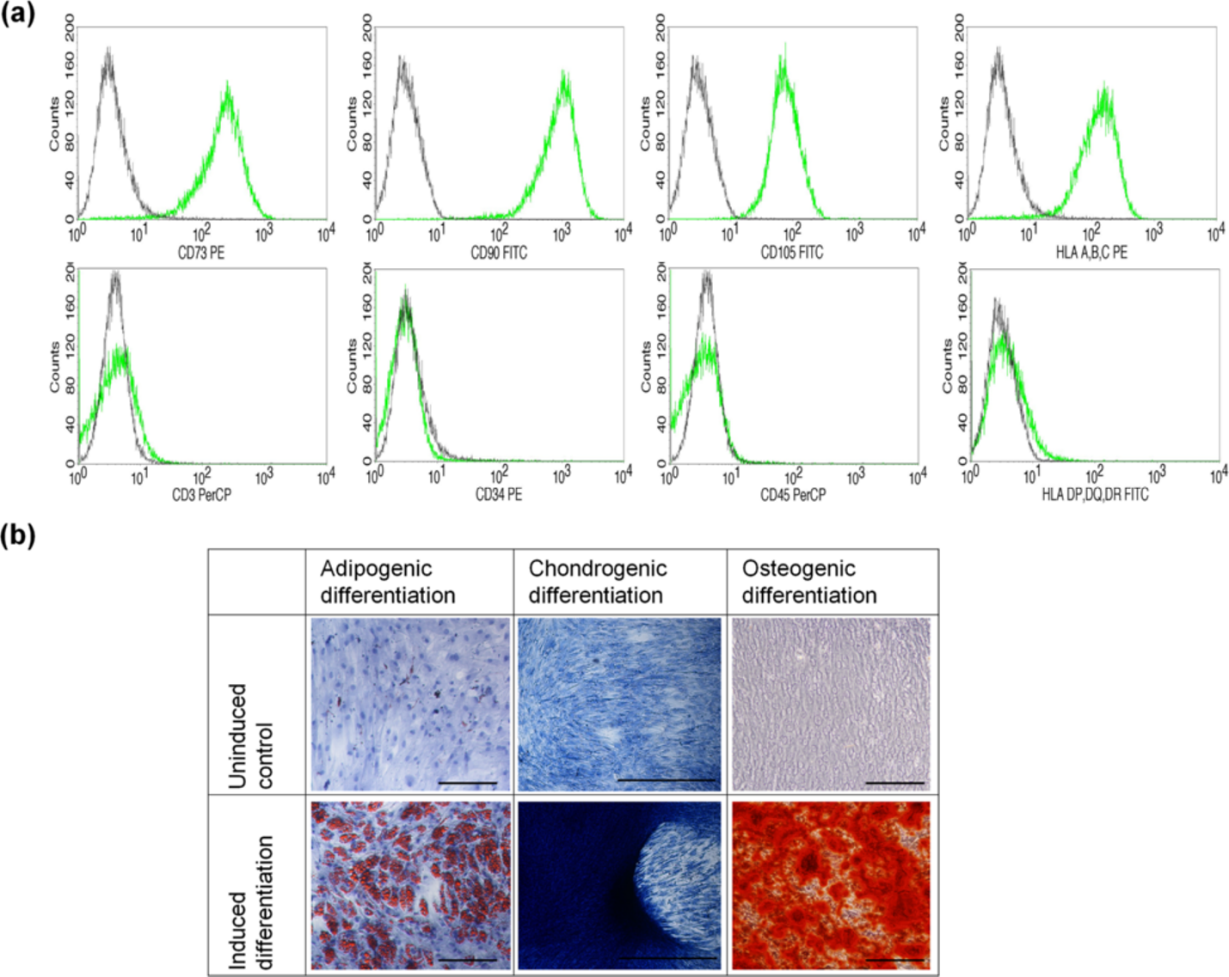
**Characterization of bone marrow stromal cells. (a)** Representative results of phenotypic characterization of good manufacturing practice-grade bone marrow stromal cells (BMSCs) by flow cytometry. More than 90% of BMSCs express CD73, CD90, CD105 and HLA A-B-C, and <5% of cells had surface antigen expression for CD3, CD34, CD45 and HLA DP, DQ, DR. **(b)** Tri-lineage differentiation capacity was confirmed. Adipogenic (Oil Red O/hematoxylin staining), chondrogenic (Alcian blue staining) and osteogenic differentiation (Alizarin red staining) could be induced, whereas no differentiation could be observed in controls without induced of differentiation. Black scale bars: 250 μm for adipogenic, 500 μm for osteogenic differentiation, and 1,000 μm for chondrogenic.

Several batches of BMSCs were isolated from bone marrow and expanded in GMP conditions using PL as a fetal calf serum alternative. After only one passage several hundred million BMSCs were consistently produced, as reported previously [6]. The BM-MNC content was 10.47 × 10^6^ ± 4.43 × 10^6^/ml. The CFU-F content was 357 ± 155 CFUs/10^6^ BM-MNCs. The CFU-F content in aspirates performed (about 3,738/ml bone marrow aspirate) is therefore well above the described range by Cuthbert and colleagues [29], confirming high-quality bone marrow aspirates were obtained. Furthermore, the doubling times of the BMSCs from each of the donors used for ectopic bone formation (Donors 1 to 5) and from the donors used for calvaria regeneration (Donors 6 to 8) are presented in Table 1. Phenotypic characterization of BMSCs by flow cytometry is presented in Figure 1a and showed that >90% of BMSCs expressed surface markers CD73, CD90, CD105 and HLA A-B-C, and <5% expressed surface markers CD3, CD34, CD45 and HLA DP,DQ,DR. Confirmation of the adipogenic, chondrogenic and osteogenic *in vitro* differentiation capacity of BMSCs is presented in Figure 1b.

**Table 1:** **Doubling times of bone marrow stromal cells used for bone formation assessment**

Donor	1	2	3	4	5	6	7	8
Passage 0 doubling time (hours)	20.8	21.5	20.4	21.8	20.2	27.0	27.8	26.4
Passage 1 doubling time (hours)	48.4	35.1	39.7	33.1	34.8	39.6	60.2	39.3
Passage 0 number of population doublings	12.1	11.2	11.7	10.9	11.7	12.4	12.1	12.6
Passage 1 number of population doublings	2.4	3.3	3.0	3.5	3.3	4.2	2.8	4.2
Cumulative population doublings	14.5	14.5	14.7	14.4	15.0	16.6	14.9	16.8
Protocol option as published by Fekete and colleagues [6]	2	2	2	2	2	1	1	1

Donors 1 to 5, ectopic; Donors 6 to 8, calvaria.

### Optimal cell dosage for bone formation

**Figure 2:**
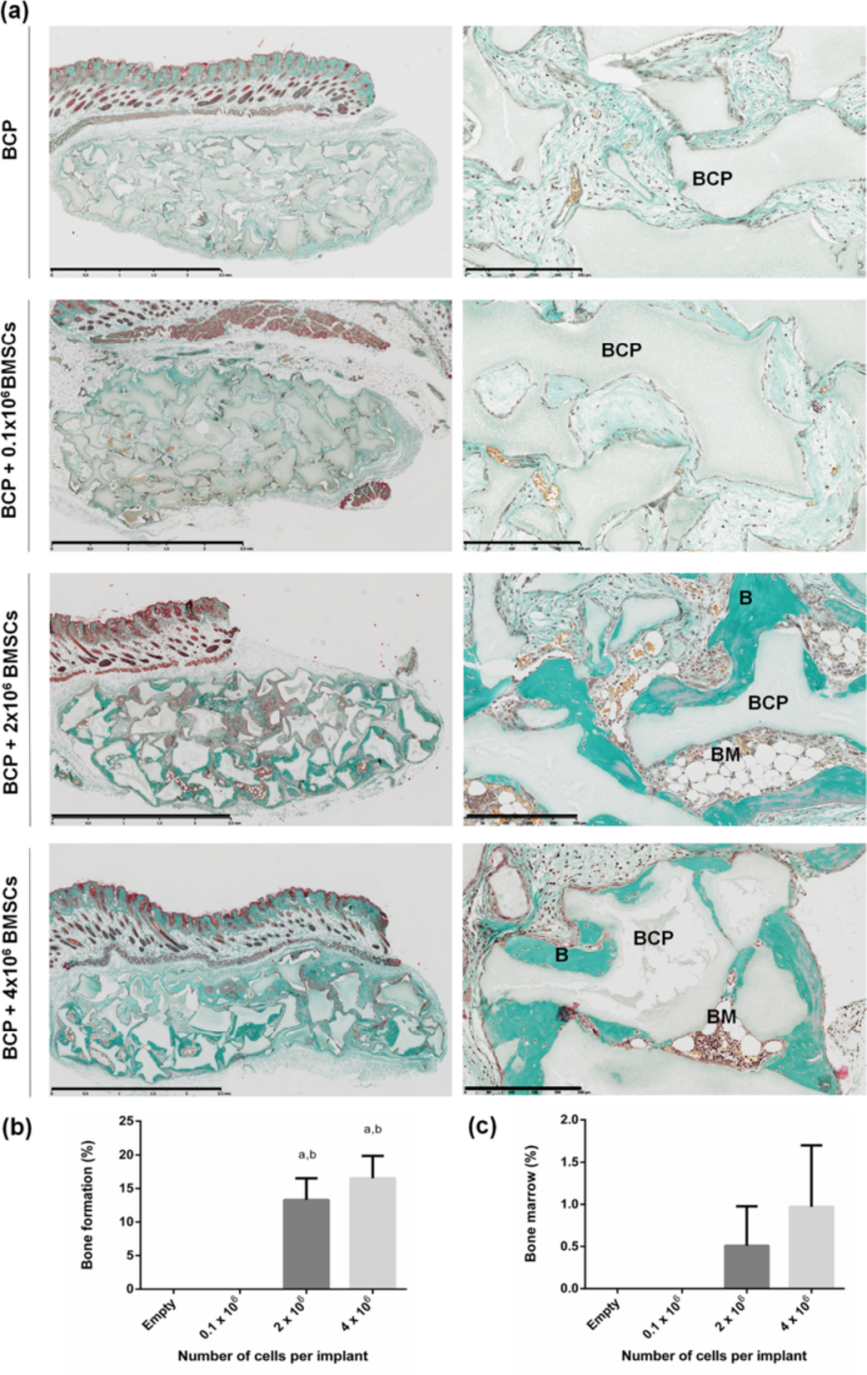
**Optimal cell dosage for ectopic bone formation. (a)** Masson trichrome staining shows biphasic calcium phosphate biomaterial (BCP, gray) in contact with newly formed bone (B, green). Mature bone marrow territories (BM) were present after 8 weeks. Scale bars: 2.5 mm and 250 μm for images in the left and right columns respectively. **(b)** Histomorphometry revealed significantly more bone in the 2 × 10^6^ to 4 × 10^6^ cell groups compared with the empty scaffolds (^a^ *P* <0.02) and compared with the 0.1 × 10^6^ cell group (^b^ *P* <0.02). **(c)** Bone marrow territories were present only in the 2 × 10^6^ and 4 × 10^6^ cell groups, but there was no statistical difference between groups. BMSCs, bone marrow stromal cells.

An initial objective of this study was to determine the number of cells required for bone formation. Different quantities of cells, in unison with BCP particles, were therefore implanted subcutaneously in nude mice. Bone and bone marrow were quantified after 8 weeks and presented as a percentage of the total implant cross-sectional area. As illustrated in Figure 2, no bone formation was demonstrated in either the 0 or and 0.1 × 10^6^ cell groups. Conversely, both the 2 × 10^6^ (13.30 ± 3.23) and 4 × 10^6^ (16.54 ± 3.33) cell groups formed significantly more bone compared with both the 0 and 0.1 × 10^6^ cell groups (*P* <0.02). There was no significant difference in the quantity of bone formed between the 2 × 10^6^ and 4 × 10^6^ cell groups. Bone marrow territories were present only in the 2 × 10^6^ (0.51 ± 0.47) and 4 × 10^6^ (0.97 ± 0.77) cell groups, but the difference between groups did not reach statistical significance. Based on these results the optimal cell dosage was set at 2 × 10^6^ cells/50 mg BCP. Since 1 cm^3^ BCP weighs 500 mg, this cell dosage translates to 20 × 10^6^ cells/cm^3^ biomaterial.

### Viability following transportation of clinical batches of fresh BMSCs

**Figure 3:**
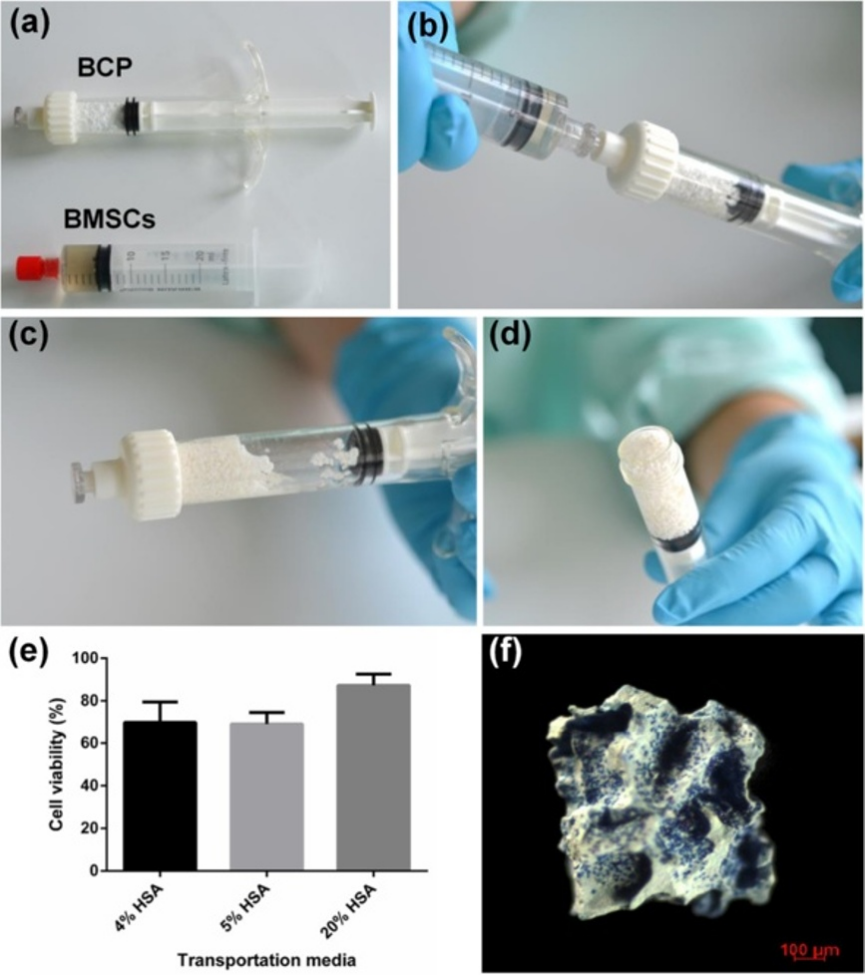
**Transportation of fresh bone marrow stromal cells. (a)** to **(d)** Syringes containing 100 million bone marrow stromal cells (BMSCs) were transported from Germany to France where they were mixed with biphasic calcium phosphate (BCP) biomaterial in sterile syringes. **(e)** No difference in viability of transported cells according to the percentage of human serum albumin (HSA) in the transportation media was found upon arrival in France. **(f)** Methylene blue staining shows cells attached to the BCP particles after 1 hour.

The determined optimal cell dosage of 20 × 10^6^ cells/cm^3^ biomaterial translates to 100 × 10^6^ BMSCs for filling clinically relevant sized bone defects of 5 cm^3^. Syringes containing 100 × 10^6^ BMSCs were shipped from Germany within 24 hours and the cell viability was determined upon arrival in France. As presented in Figure 3, the average percentage cell viability upon arrival in syringes with 4% HSA was 69.9 ± 9.55%, with 5% HSA was 69.15 ± 5.43% and with 20% HSA was 87.27 ± 5.26%, with no statistical difference between transportation media. Syringes containing the BMSCs and biomaterial are shown in Figure 3a,b,c,d. A standard operating procedure was developed for mixing cells and biomaterial under aseptic condition in the surgical room for 1 hour. It was previously demonstrated that 45 to 50% of BMSCs suspended in HSA attached to BCP particles after 1 hour [17]. Although we found that the maximal cell attachment was attained at approximately 4 hours after seeding, a 1-hour attachment time was chosen for the preclinical and clinical trials because this time frame is appropriate in the surgical setting. As shown by methylene blue staining (Figure 3f), this procedure guaranteed adequate attachment and even distribution of BMSCs on the BCP granules prior to implantation.

### Ectopic bone formation following transportation of several batches of BMSCs

**Figure 4:**
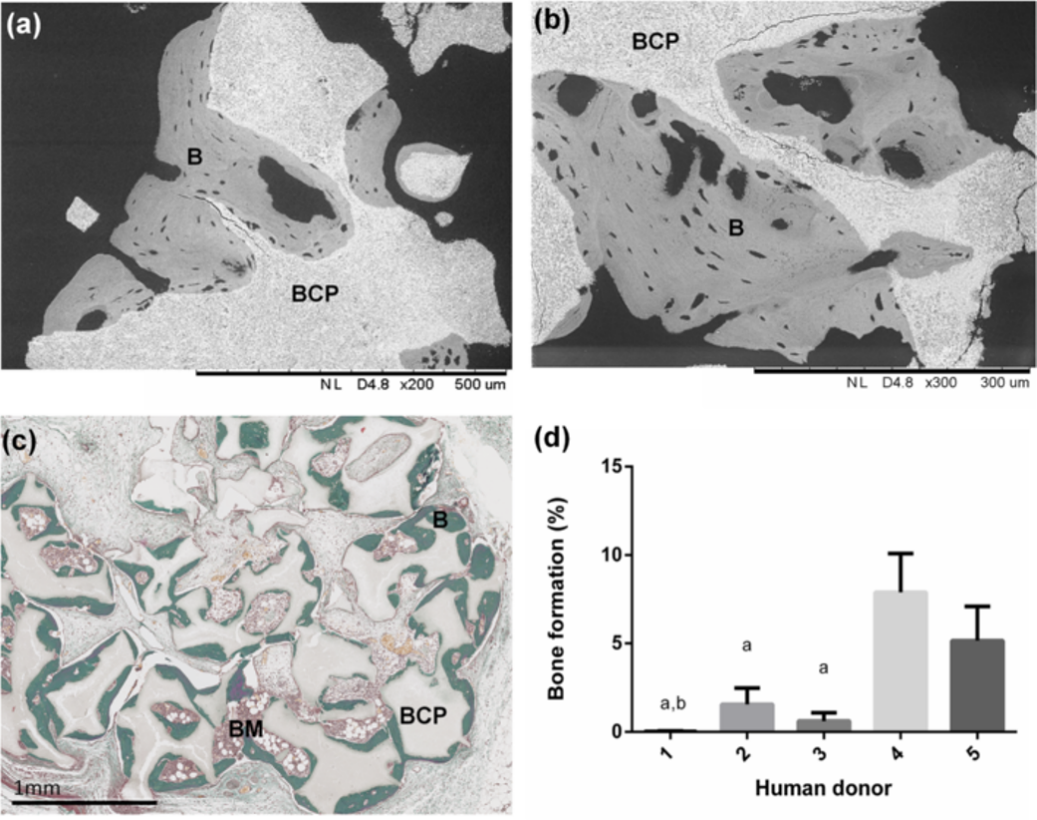
**Ectopic bone formation of freshly transported batches of bone marrow stromal cells. (a)**, **(b)** In backscattered electron imaging, the biphasic calcium phosphate (BCP) ceramic and mineralized bone can be differentiated by their relative gray densities. The BCP particles were surrounded by a well-mineralized lamellar bone tissue in the BCP + BMSCs group after 8 weeks. **(c)** Masson trichrome staining showed bone formation (B) at the periphery of the explants that contained bone marrow compartments (BM). **(d)** Histomorphometry of Masson trichrome staining sections revealed significant differences in the bone induction capacity between human donors. ^a^Statistically lower compared to Donor 4 (*P* <0.05). ^b^Statistically lower compared with to Donor 5 (*P* <0.05). BMSCs, bone marrow stromal cells.

The ectopic model was performed to demonstrate the osteoinduction of the BMSC/BCP combination of seven GMP batches from five human donors that were transported fresh. The subcutis implantations in nude mice showed a thin fibrous tissue capsule with abundant vascularization without sign of tissue necrosis, local inflammation or infection. After 8 weeks, no ectopic bone formation was observed in control groups with BCP biomaterial alone (data not shown). In contrast, in the BCP + BMSCs group, ectopic bone formation was observed at the periphery of the explants and demonstrated a well-mineralized lamellar bone tissue with osteocyte lacunae, as illustrated by back-scattered electron microscopy and Masson trichrome staining in Figure 4a,b,c. The percentage of bone formation revealed significant differences in the bone induction capacity between human donors, as presented in Figure 4d.

### Regeneration of critical size defects in calvaria of nude mice

**Figure 5:**
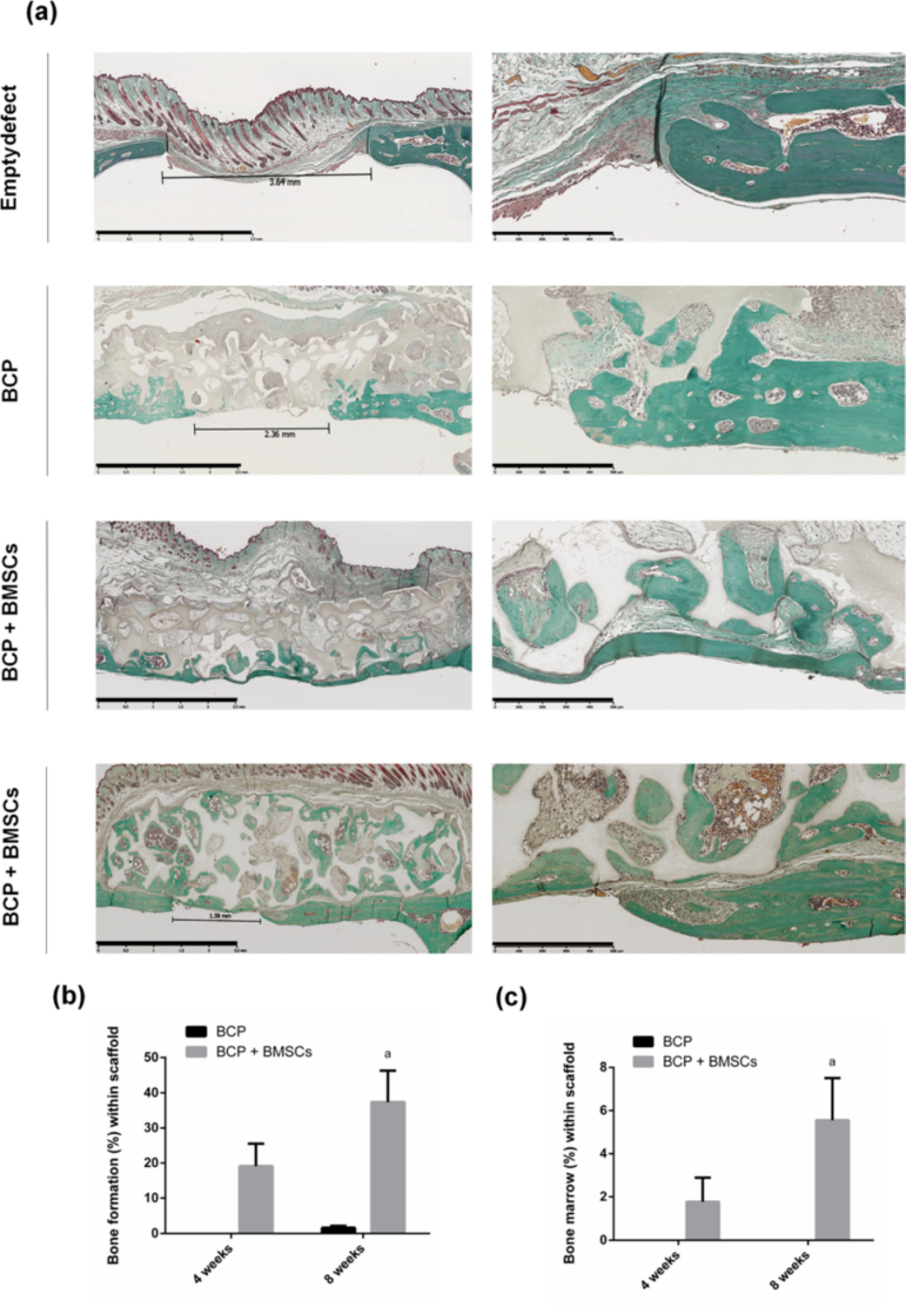
**Regeneration of critical size defects in calvaria of nude mice. (a)** Masson trichrome staining (MT) of calvaria defects after 8 weeks shows bone in green and biphasic calcium phosphate (BCP) in gray. Scale bars: 2.5 mm and 500 μm for images in the left and right columns respectively. **(b)**, **(c)** Histomorphometry of MT sections revealed that there was significantly more (^a^ *P* <0.05) newly formed bone and bone marrow formed with the scaffolds of the BCP + BMSCs group compared with BCP discs without cells. BMSCs, bone marrow stromal cells.

To investigate the potential of BMSCs to regenerate bone defects, BMSCs were implanted with BCP discs over critical-sized cranial defects. Defects that were either left empty or overlain with BCP biomaterial alone served as controls. All animals recovered well from surgery without signs of neurological damage. As shown in Figure 5a,b,c, no statistical difference in percentage of bone or bone marrow formation within the BCP discs was found between the BCP and BCP + BMSCs groups 4 weeks after implantation; however, a trend towards increased bone formation in the BCP + BMSCs group was observed (*P* <0.088). After 8 weeks, the percentage of bone formation was significantly higher in scaffolds with BMSCs (37.41 ± 8.93%) than BCP scaffolds alone (1.58 ± 0.51%) (*P* <0.02; Figure 5b). Likewise, bone marrow presence was higher in the BCP + BMSCs group after 8 weeks (5.56 ± 1.95%) compared with the BCP group (no bone marrow presence) (*P* <0.05; Figure 5c). Empty defects failed to achieve bone defect closure (percentage of defect closure was 26.29 ± 14.29% after 4 weeks and 26.97 ± 9.53% after 8 weeks) and were filled with fibrous tissue, confirming that the model was a critical-sized bone defect. Defect closure with BCP scaffolds alone was 35.75 ± 5.08% after 4 weeks and 45.25 ± 7.01% after 8 weeks. Some BCP + BMSCs samples achieved complete defect closure, as shown in Figure 5a. Most, however – especially those with significant bone formation within the biomaterial scaffold – did not achieve complete defect closure; rather, a bone bridging was accomplished by the bone-filled scaffold overlying the defect. Defect closure of BCP scaffolds with BMSCs was 40.50 ± 11.01% after 4 weeks and 55.15 ± 12.09% after 8 weeks; this was not statistically higher than the other groups however.

### Bioluminescence imaging of luciferase-expressing BMSCs

**Figure 6:**
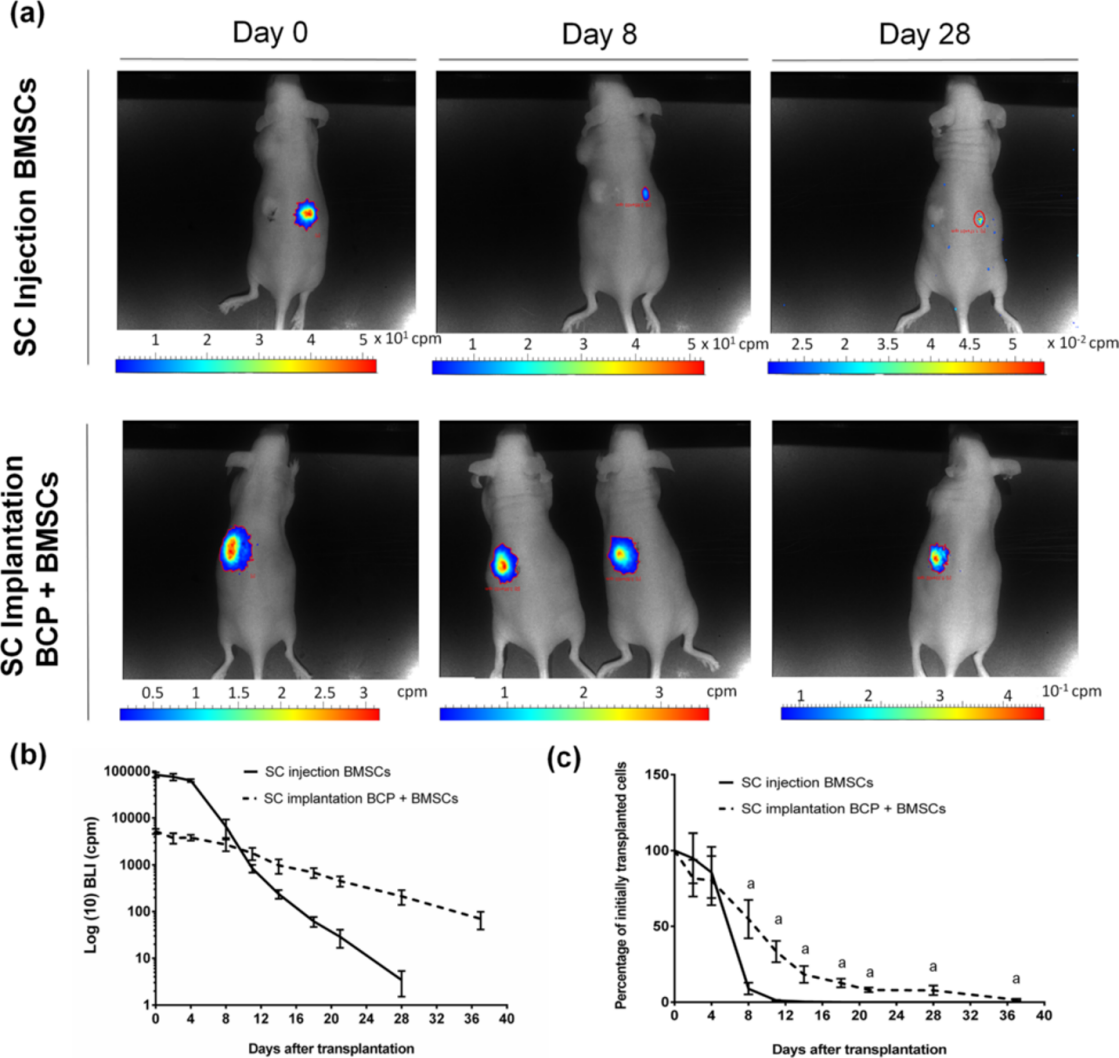
**Bioluminescence imaging of luciferase-expressing bone marrow stromal cells. (a)** Representative bioluminescence imaging (BLI) for a control group that received biphasic calcium phosphate (BCP) alone on their left dorsal side and subcutaneous (SC) injections of bone marrow stromal cells (BMSCs) on their right dorsal side, and the experimental group that received subcutis implantations of BCP + BMSCs. **(b)** The BLI signal, expressed as counts per minute (cpm), decreased after transplantation, while BCP dampened the BLI signal. **(c)** When the number of remaining cells compared with the number of initially transplanted cells was expressed at each time point, SC implantation of BCP + BMSCs was significantly higher from day 8 onwards compared with the SC injections group (^a^ *P* <0.01). Approximately 1.5% of the initial number of transplanted cells in the SC BCP + BMSCs group remained at day 37.

To assess the survival capacity and biological activity of BMSCs following implantation *in vivo*, BLI was performed on Luc/eGFP MSCs transplanted in nude mice. As depicted in Figure 6a,b, in the subcutaneous injection control group the BLI signal was 63,371.43 ± 4,396.13 at day 4, after which the signal dramatically decreased: at day 28 only 3/6 mice had a detectable BLI signal (3.41 ± 1.90) and no signal was present at day 37. In the experimental group (BCP + BMSCs), the BLI signal also reduced throughout the duration of the experiment; however, it was a much less striking decline compared with the subcutaneous injection control group, as seen in Figure 6b. BCP particles dampen the BLI signal, as evidenced from Figure 6b, since the same quantity of cells was transplanted into both groups at day 0 but the BLI signal in the BCP + BMSCs group was significantly lower than that of the subcutaneous injection group (5,184 ± 622.96 vs. 84,082.61 ± 10,478.04, *P* <0.05). Figure 6c illustrates the percentage of the original signal at day 0 remaining at each time point. At day 8 there was a significantly higher percentage of initially transplanted cells remaining in the BCP + BMSCs group, compared with the subcutaneous injection group (54.72 ± 12.69 vs. 8.89 ± 3.78, *P* <0.01). This observation was corroborated at every later time point. By day 37, 1.57 ± 0.63% of the initial BLI signal remained in the BCP + BMSCs group, while there was no signal remaining in any of the subcutaneous injection mice.

### Identification of human cells by *in situ*hybridization

**Figure 7:**
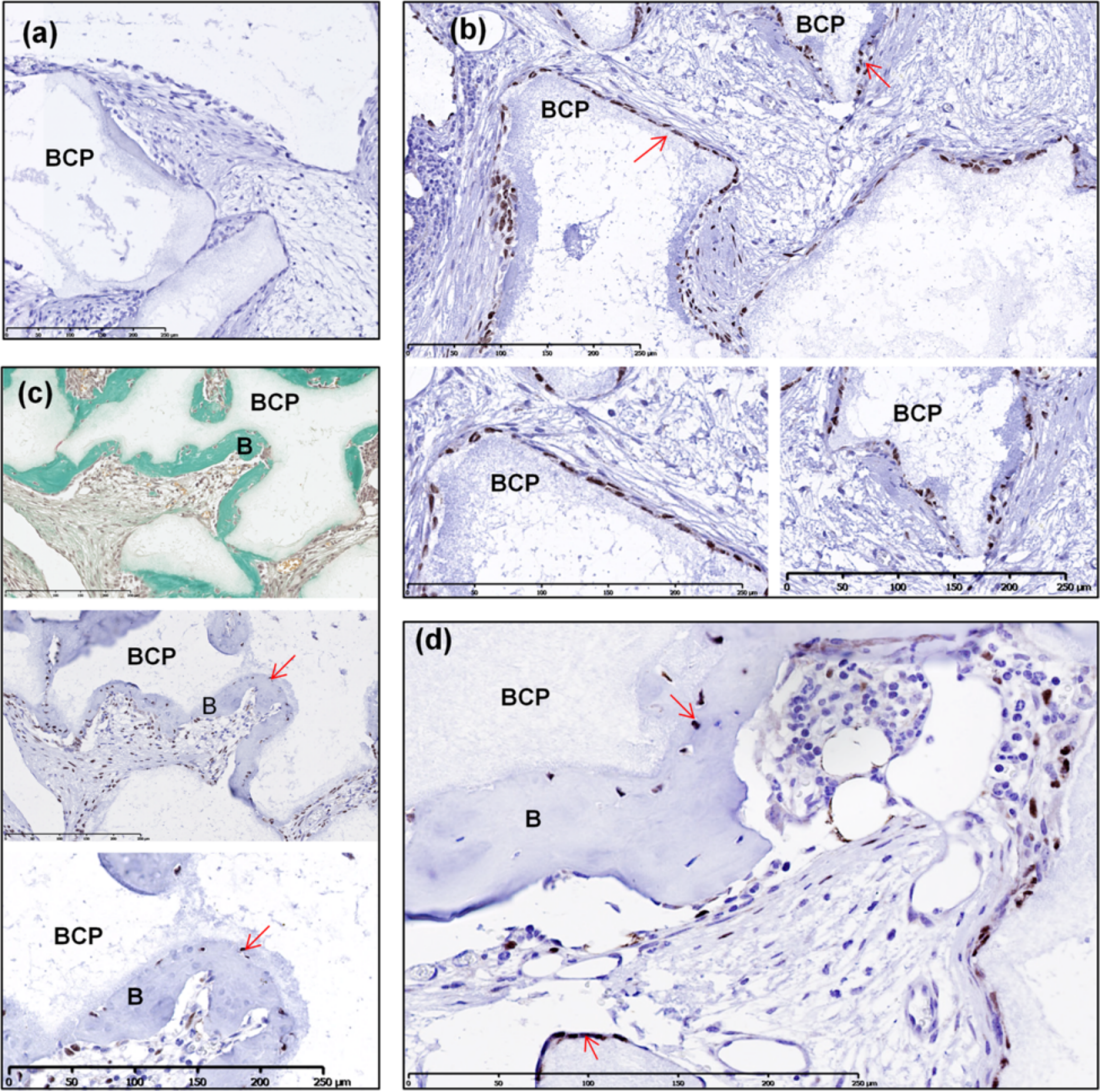
***In situ*** **hybridization using the human-specific repetitive** ***Alu*** **sequence for identification of human cells.** All samples were analyzed after 8 weeks of subcutaneous implantation in nude mice **(a)** Negative control. **(b)** Although explants were composed primarily of mouse cells (purple nuclei), human cells (brown/black) were identified attached along the periphery of biphasic calcium phosphate (BCP) particles (red arrows). These regions of interest are shown in magnified view. **(c)** Masson trichrome staining of explants with a serial section showing human cells present (red arrow), with a magnified view also presented. **(d)** Explant showing mice cells (purple nuclei) and human cells (brown/black nuclei) embedded in osteocyte lacunae and surrounding BCP particles (red arrows). Scale bars: 250 μm. B, bone formation.

*In situ* hybridization using the human-specific repetitive *Alu* sequence allowed for the identification of human cells in explants. As evidenced in Figure 7, after 8 weeks of implantation, it was observed that while most of the explants were comprised of host cells (purple nuclei), there were also human cells present (brown/black nuclei). Few human cells were present in the fibrous tissue, while more human cells were identified attached along the periphery of BCP particles. Furthermore, human osteocytes were found embedded in lacunae dispersed throughout the bone matrix with osteocytes of mice origin in close proximity.

## Discussion

Repair of bone defects remains a significant clinical challenge. In this study, 100 million BMSCs were transported in each syringe from a GMP facility in Germany to another site in France where they were mixed with biomaterial and implanted, mimicking the clinical scenario where large bone defects shall be regenerated. Bone containing mature bone marrow territories was formed in ectopic sites and in calvaria defects. Following implantation, the human cell number was drastically reduced; nevertheless, by 8 weeks some human cells were observed in osteocyte lacunae in newly formed bone.

It was important to investigate the optimal cell dosage for implantation in this study since, for clinical relevance, implanting too few cells would yield inadequate bone healing, while implanting more cells than required would result in cell wastage and more prolonged *in vitro* expansion times. A sharp increase in bone formation was found from 0.1 × 10^6^ cells per implant to 2 × 10^6^ cells per implant. Interestingly, doubling the cell density to 4 × 10^6^ cells did not yield significantly more bone or bone marrow territories, demonstrating that a threshold was reached beyond which more implanted cells did not yield more bone formation. This result is in agreement with a previous study [12]. The ratio of cells to weight of biomaterial was then scaled up to a clinically equivalent dosage of 100 × 10^6^ BMSCs per 5 cm^3^ of BCP granules. A standard operating procedure for mixing cells and biomaterial before implantation was developed. Bone formation was evaluated at 8 weeks since it has been shown previously that there is only minimal bone formation prior to this duration [12].

The percentage of HSA in the transportation media was reduced from 20% because it favored the formation of cell aggregates. The rationale for including both 4% and 5% HSA was because 4% HSA is commercially available in France whereas 5% HSA is commercially available in Germany. Due to the large heterogeneity between human donors, it was not possible to assess whether the HSA concentration affected bone formation. However, independently of the percentage of HSA, the cell viability after transportation was approximately 70% and it is expected that this would be even higher in the clinical scenario due to the closer proximity of GMP production sites to the operating room. Cells attached to the BCP scaffold after only 1 hour and this is a clinically compatible preparation time for surgery of patients. Cells were transported at 20°C in this study; however, it must be noted that 4°C was since found to be a more optimal temperature [17].

Mature bone, including the presence of bone marrow territories, was observed after 8 weeks in subcutis explants. Hematopoiesis was only observed in explants with significant bone formation. This timing is well in agreement with other reports describing ectopic bone formation [18, 19]. The transplants devoid of implanted BMSCs did not achieve any bone formation. Furthermore, this study demonstrated that human BMSCs could form bone and aid healing of critical-sized calvaria defects after 8 weeks. Abundant bone was formed within the BCP discs with mature bone marrow territories, which is relevant for maxillofacial regeneration.

The significant variability in the bone formation capacity between human donors is in agreement with previous studies [30, 31]. Age, gender, disease and medications have been associated with altered prevalence of BMSCs in bone marrow as well as their activity *in vitro*[32–34]. In the current study, BMSCs were attained from healthy human donors ranging in age from 21 to 26, so age or disease was not a factor here. In addition, there was no correlation found between the bone formation capacity of the BMSCs from the five donors used for ectopic bone formation and the colony-forming efficiency of the bone marrow aspirates, expressed as CFUs-F/10 × 10^6^ MNCs (data not shown). Others found that there was no correlation between gender and bone formation capacity [30]. Understanding the underlying factors responsible for donor variability is of paramount importance for the clinical application of BMSCs for bone regeneration and will be the focus of future investigations.

In this study, the fate of the implanted cells in subcutaneous sites in the early and late stages of engraftment was evaluated using both bioluminescence imaging and *in situ* hybridization for the human-specific *Alu* sequence. We observed significant loss of the BLI signal over the experiment duration, with only approximately 1.5% of transplanted cells remaining after 37 days. It must be noted, however, that since the Luc/eGFP MSCs used for the bioluminescence imaging were in passage 8, this could have possibly contributed to their early disappearance due to higher doubling times that accompany high passage numbers. Nevertheless, this finding is in agreement with other studies noting a large loss of transplanted BMSCs [24, 25]. Furthermore, although the BCP particles reduced the BLI signal intensity of BMSCs, the reliability of BLI for determining viable cell numbers was previously confirmed with BCP and BMSCs [24].

At 8 weeks, using *in situ* hybridization for the human-specific *Alu* sequence (BMSCs implanted at passage 2), it was observed that there were some human cells found along the periphery of the BCP particles and embedded in osteocyte lacunae; however, the vast portion of cells present were of host origin. A previous study also found donor cells present in newly formed bone tissue [13]. However, in other studies no implanted BMSCs were observed in newly formed bone and host cells were directly responsible for bone formation [23, 35, 36]. There could be numerous reasons for these conflicting observations between studies, including the methods of *ex vivo* expansion (fetal calf serum vs. PL), the type of scaffold used (some scaffolds are better than others at retaining BMSCs), and even the strain of recipient mice (how immune deficient they are). The primary factors responsible for the large cell death of transplanted BMSCs include the ischemic environment [25] and the lack of glucose that the BMSCs encounter [37]. Despite this substantial cell death, the transplanted BMSCs mediated bone formation by host BMSCs, suggesting that transplanted BMSC-secreted paracrine molecules may play an important role in bone formation by host BMSCs.

It has been shown previously that BMSC modulation of the foreign body reaction and interaction with the host immune cells play a role in BMSC-mediated bone formation [36]. Furthermore, we have recently demonstrated the role of BMSCs as mediators rather than effectors of bone formation by impacting the foreign body reaction [38]. BMSCs attracted circulating hematopoietic stem cells and prompted their differentiation into macrophages M1 and osteoclasts; ablation of osteoclastogenesis largely inhibited BMSC-mediated bone formation [38]. However, at present, the precise mechanisms of how donor BMSCs recruit host BMSCs to the site is uncertain and warrants further investigation.

In this study, BMSCs have been successfully expanded, transported and implanted for bone repair, mimicking a clinically relevant scenario. In unison with BCP biomaterial, BMSCs achieved ectopic bone formation with bone marrow territories and successfully repaired critical-sized cranial defects. A drastic loss of BMSC viability after transplantation was observed. Newly formed bone contained some cells of human origin, but explants were primarily composed of host cells. This study demonstrates the safety and efficacy of BMSC/BCP combinations for bone regeneration.

## Conclusions

Our findings provide therapeutic evidence that BMSC/BCP associations may be used for bone regeneration and present crucial information for the implementation of human BMSC therapy in clinical practice for the treatment of nonunion fractures.

## Abbreviations

BCP: biphasic calcium phosphate
BLI: bioluminescence imaging
BM-MNC: bone marrow mononuclear cell
BMSC: bone marrow stromal cell
CFU-F: colony-forming unit
GFP: green fluorescent protein
GMP: good manufacturing practice
HSA: human serum albumin
PL: plate lysate.
